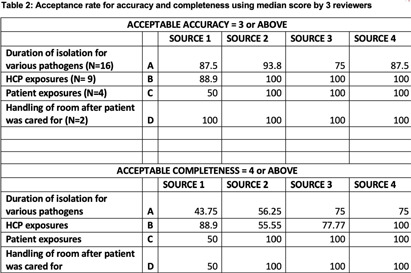# Can Artificial Intelligence Support Infection Prevention and Control Consultations?

**DOI:** 10.1017/ash.2024.137

**Published:** 2024-09-16

**Authors:** Natalie Ross, Karen Brust, Takaaki Kobayashi, Oluchi Abosi, Jorge Salinas, Alexandra Trannel

**Affiliations:** University of Iowa Hospitals & Clinics; Stanford University

## Abstract

**Background:** Artificial intelligence (AI) tools have demonstrated success in US medical licensing examinations; however, their utility in infection prevention and control (IPC) remains unknown. **Methods:** The program of hospital epidemiology handles consultation calls and records each question and answer. Using 2022 data, we selected 31 frequently asked questions. We utilized four AI tools, including Chat GPT-3.5 and 4.0, Bing AI, and OpenEvidence, to generate answers. We predefined scales (Table 1) to capture responses by three reviewers, including two hospital epidemiologists and one infection preventionist. The mean score of ≥ 3 and ≥ 4 was considered acceptable in accuracy and completeness, respectively. We reported the percentage of responses with acceptable accuracy and completeness out of assessed questions for each category. **Results:** Among 31 questions, 16 were associated with isolation duration, 9 with healthcare personnel (HCP) exposure, 4 with cleaning contaminated rooms, and 2 with patient exposure. Regarding accuracy, most AI tools performed worse in questions about isolation duration, ranging between 75% and 93.8%. All AI tools, except OpenEvidence, had a 100% accuracy rate for HCP and patient exposure. All AI tools had a 100% accuracy rate for contaminated room handling. The highest overall acceptable accuracy rate was observed in Chat GPT-3.5. Regarding completeness, most AI tools performed worse in questions about isolation duration, ranging between 44% and 75%. All AI tools, except OpenEvidence, had a 100% completeness rate for contaminated rooms and patient exposure. The highest overall acceptable completeness rate was observed in Bing AI (Table 2). **Conclusions:** All AI tools provided reasonable answers to commonly asked IPC-related questions, although, there were variations among different tools used. AI could be used to supplement the infection control program, especially if resources are limited.

**Figure:**
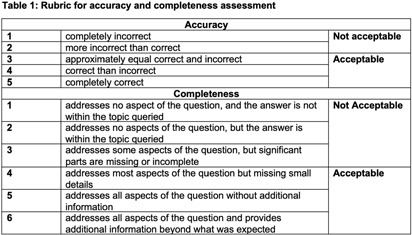

**Figure:**